# Enhanced acupuncture therapy for radiotherapy-related neuropathic pain in patients with gynecologic cancer: a report of two cases and brief review

**DOI:** 10.3389/fneur.2023.1163990

**Published:** 2023-06-07

**Authors:** Zhou Dan-feng, Rong Jian-cheng, Zheng Shu-zhen, Zhang Kun, Yang Hong-zhi, Yang Lian-sheng, Tang Chun-zhi

**Affiliations:** ^1^Department of Acupuncture and Moxibustion, The Third Affiliated Hospital of Sun Yat-sen University, Guangzhou, China; ^2^Department of Rehabilitation, Jiangmen Central Hospital, Jiangmen, China; ^3^Department of Traditional Chinese Medicine, The Third Affiliated Hospital of Sun Yat-sen University, Guangzhou, China; ^4^Medical College of Acu-Moxi and Rehabilitation, Guangzhou University of Chinese Medicine, Guangzhou, China; ^5^South China Research Center for Acupuncture and Moxibustion, Medical College of Acu-Moxi and Rehabilitation, Guangzhou University of Chinese Medicine, Guangzhou, China

**Keywords:** neuropathic pain, radiotherapy, acupuncture, cancer, case report

## Abstract

As radiation therapy is increasingly utilized in the treatment of cancer, neuropathic pain (NP) is a common radiotherapy-related adverse effect and has a significant impact on clinical outcomes negatively. However, despite an improved understanding of neuropathic pain management, pain is often undertreated in patients with cancer. Herein, we reported two cases with radiotherapy-related neuropathic pain (RRNP) who presented a positive reaction to acupuncture. Patient 1 (a 73-year-old woman) with gynecologic cancer complained of burning and electric shock-like pain in the lower limb after radiotherapy. With the accepted combination of acupuncture and drugs, the pain was alleviated completely in 8 weeks. Patient 2 (a 64-year-old woman) accepted acupuncture in the absence of medication because of her inability to tolerate the adverse events of anticonvulsant drugs. She achieved remission of pain 4 weeks later. The results of this study showed that acupuncture might be promising for controlling the RRNP in patients with cancer, especially who were intolerant or unresponsive to medications.

## 1. Introduction

Neuropathic pain (NP) is defined as “pain caused by a lesion or disease affecting the somatosensory system” (1), which is different from nociceptive pain due to damage to the non-neural tissue and activation of nociceptors. In patients with cancer, it had been reported that NP affects ~20–40% of populations (2) and severely interferes with emotional and physical function, influences the quality of life negatively, as well as is associated with the clinical outcome directly (3, 4). In etiology, cancer-related NP could be subdivided into tumor-related (caused by tumor *per se*) and treatment-related NP (caused by treatments, such as chemotherapy or radiotherapy) (5). At present, pain secondary to tumor have been universally appreciated, and the neurotoxicity of chemotherapy is increasingly recognized as well. In comparison, there exists a sparsity of direct data focused on radiotherapy-related neuropathic pain (RRNP) (6) despite the increased utilization of radiotherapy in cancer care and the overall increase in cancer survivorship.

Current analgesic approaches are pharmaceutical (including antidepressants, anticonvulsants, and opioids) and non-pharmaceutical. Despite an improved understanding of pain management and many approaches, pain is often under-treated in patients with cancer (7). A system review revealed that approximately one-third of patients with cancer do not receive appropriate analgesia proportional to their pain intensity (8). Therefore, for optimal pain control, a multimodal approach of medications and non-pharmaceutical therapy could be applied in conjunction with disease-directed treatment (9), especially for patients who show no positive response to these conventional treatments.

As a therapy of traditional Chinese medicine, acupuncture is widely used for palliative and supportive care for cancer patients, and evidence is increasing over the years (10). Some studies suggested that acupuncture might be effective for chemotherapy-induced neuropathic pain (CINP) (11–14). Based on the similar concept of acupuncture, acupuncture might have potential in the pain management of RRNP. Herein, we report the successful application of acupuncture for the management of RRNP in patients with gynecologic cancer. The article follows the CARE guidelines (15).

## 2. Case presentation

### 2.1. Case 1

**Figure 1:**
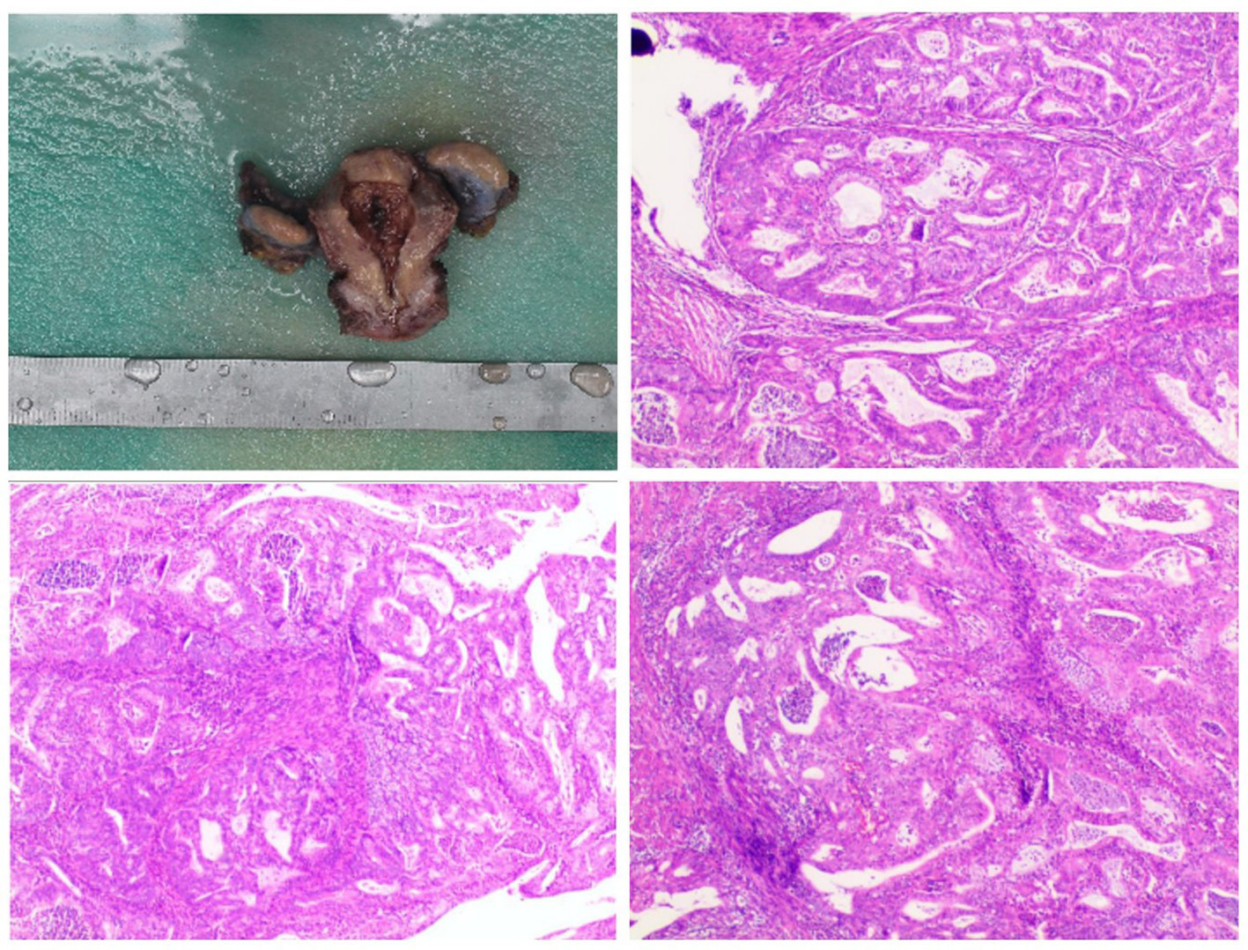
Pathological morphology examination.

**Figure 2:**
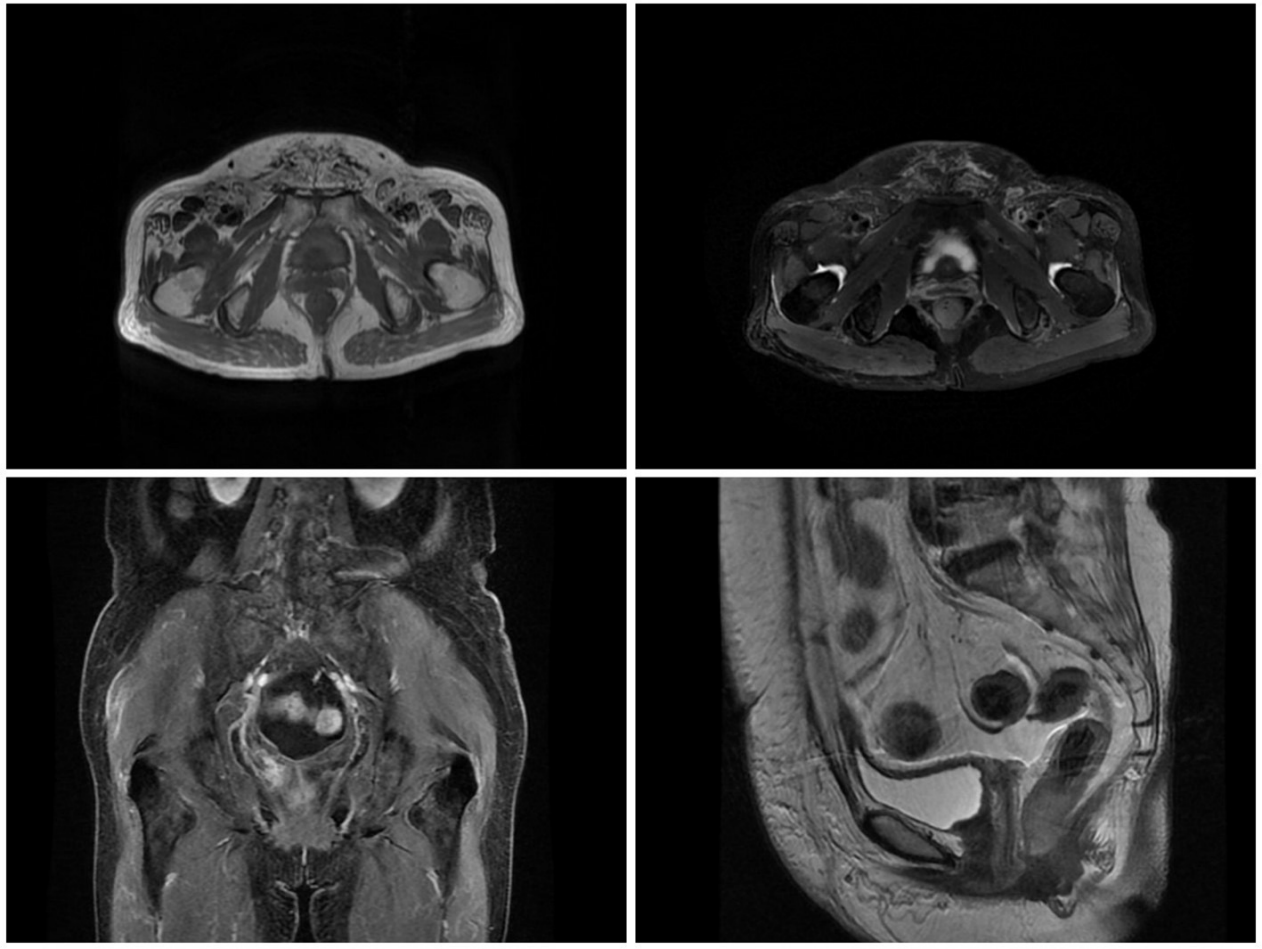
The pelvic MRI on 1 month after surgery showed no residual tumor tissue or metastasis.

**Figure 3:**
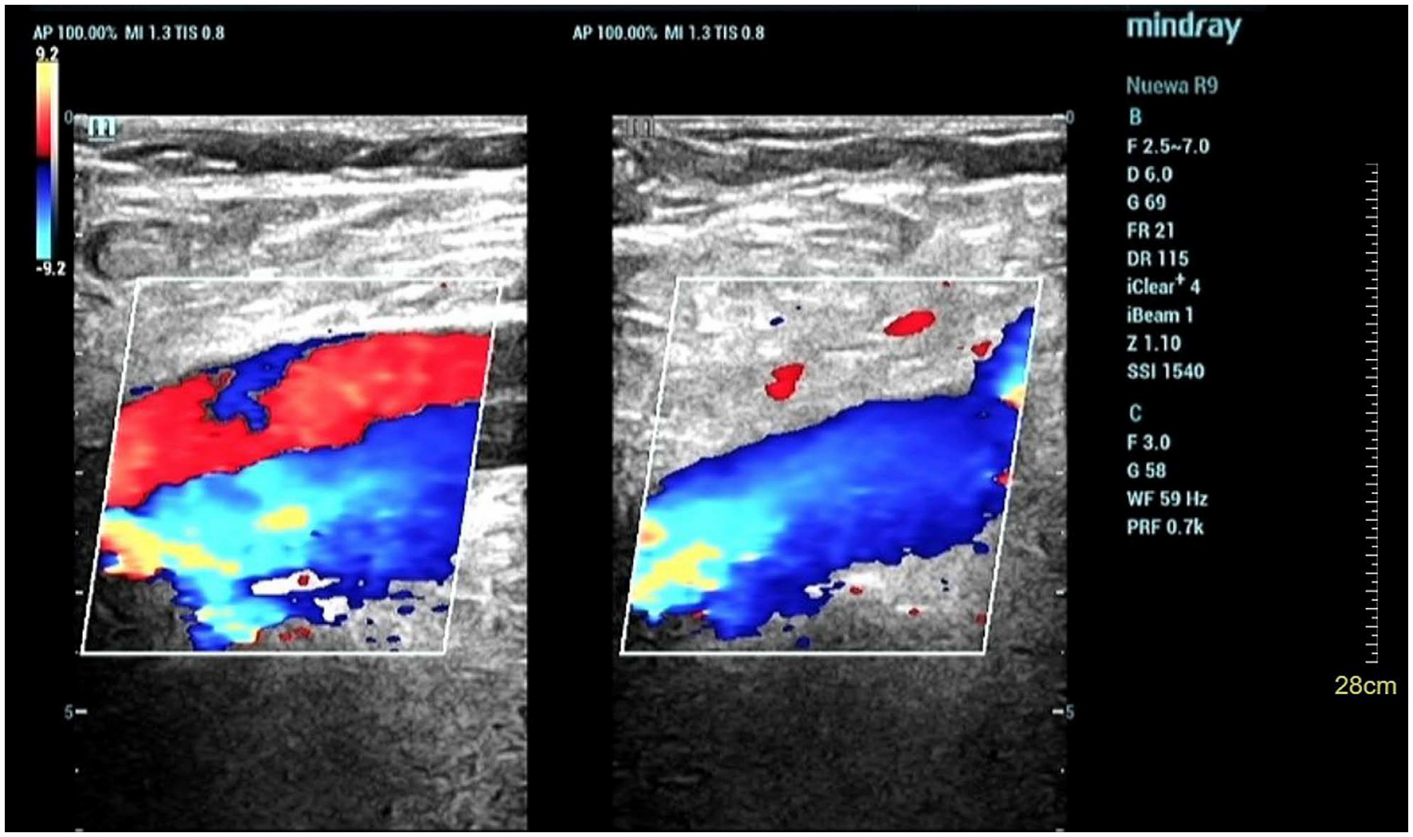
Ultrasonic testing showed venous blood flow in the lower extremities is smooth.

A 73-year-old woman who presented with postmenopausal bleeding for 20 years was admitted to the gynecology department at the Third Affiliated Hospital of Sun Yat-Sen University. Her past medical history revealed type 2 diabetes mellitus with regular hypoglycemic drugs. On admission, the test of serum tumor markers showed that carbohydrate antigen-125 was 90.4 U/ml, carbohydrate antigen-199 was 41.6 U/ml, and human epididymal protein 4 was 153 mmol/L. Considering the results of the pathological morphology examination (Figure 1) comprehensively, she was diagnosed with endometrial carcinoma. On the 10th day after admission, the gynecologist performed a hysterectomy plus lymph node dissection for her. After 1 week of surgery, she accepted chemotherapy (docetaxel 100 mg, carboplatin 500 mg, 1 day) and was discharged. The pelvic magnetic resonance imaging (Figure 2) at 1 month after surgery showed no residual tumor tissue, or metastasis was detected. Hence, she started to accept radiotherapy (IMRT PTV:50Gy/2Gy/25F). Approximately 3 months after surgery, she was back to the hospital because of the burning, electric shock-like pain, and pitting edema of the left lower limb that got an outbreak. Other complaints included constipation, frequent micturition, and insomnia. Neoplasm metastasis, recurrence, and deep venous thrombosis were excluded through a series of examinations like pelvic imaging and ultrasonic testing (Figure 3). She accepted oral pain relief drugs (pregabalin 75 mg Bid and mecobalamin 0.5 mg Tid) for 2 weeks but her symptoms did not improve. Therefore, she sought the aid of acupuncture in the outpatient department ~4 months after surgery. Physical examination showed hyperalgesia in the medial side of the lower limb, pressing pain (++) in the left groin area, and weakness of muscle strength of the left lower extremity. The pain intensity score evaluated by the numerical rating scale (NRS) was as high as 7 points. To control the pain better, we applied enhanced acupuncture therapy, accompanied by adjusting the dosage of medication (pregabalin 75 mg 8 am/150 mg 8 pm). The acupuncture treatment procedure was composed of manual acupuncture and electro-acupuncture, which was given 3 times per week, a total of 20 sessions of treatments within 8 weeks and implemented by an experienced and certified acupuncturist who had over 5 years of experience. The details of acupuncture based on the theory of traditional Chinese medicine, such as the acupoints, we chose and needling methods are demonstrated in Supplementary material 1.

**Figure 4:**
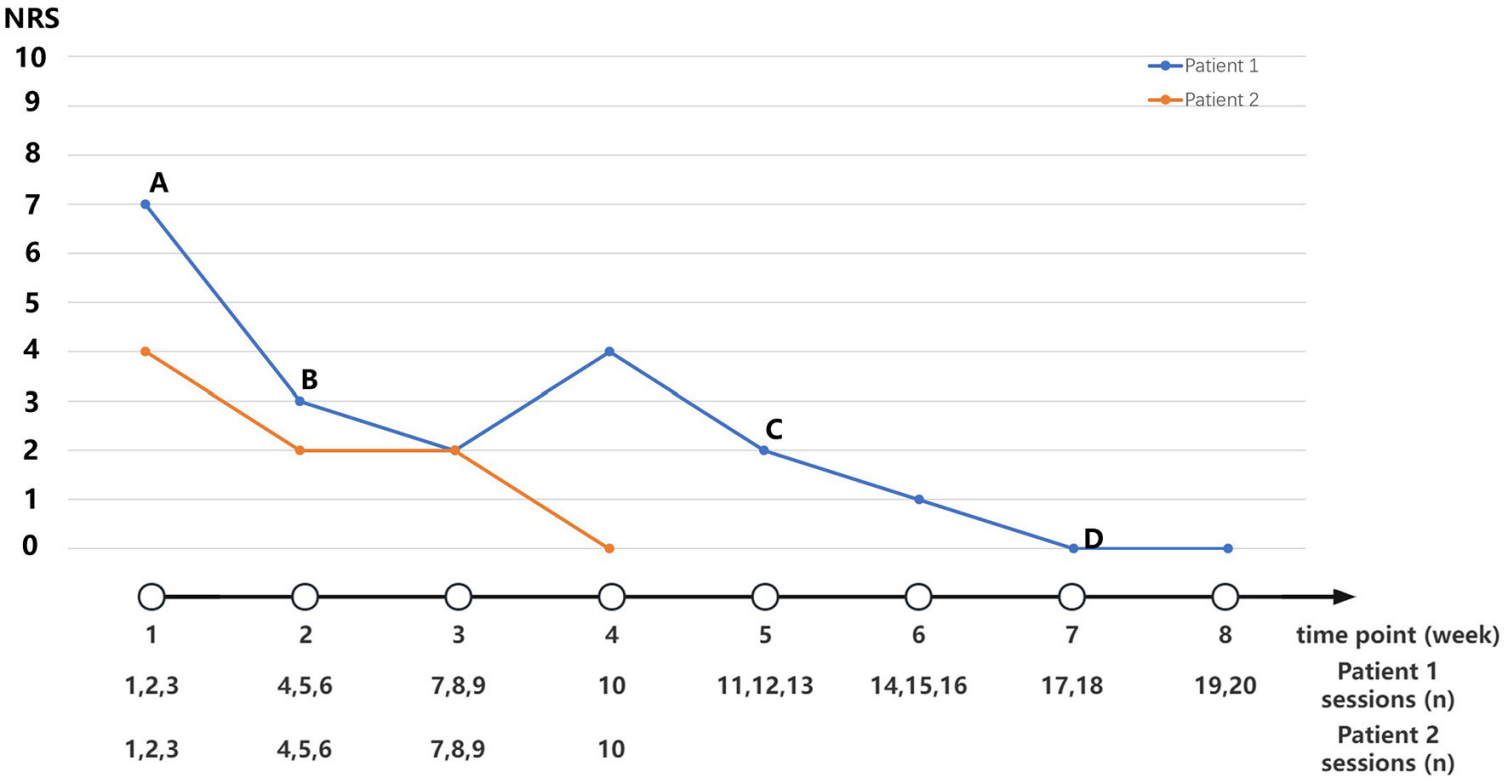
Medication adjustment and NRS scores along the timeline of disease. **(A)** Pregabalin 225 mg/day (75 mg 8 am, 150 mg 8 pm). **(B)** Pregabalin 150 mg/day (75 mg Q12 h). **(C)** Pregabalin 75 mg/day (75 mg 8 pm). **(D)** Stop medication intervention.

After five times of performing therapies, the patient claimed that the pain had relief significantly, and the NRS scores decreased from 7 to 3. Subsequently, accompanied by acupuncture therapy and the improvement of pain, the dose of pregabalin was reduced gradually until it was discontinued (Figure 4). After 20 times of therapy, the patient felt relieved of the pain completely, but the edema of the lower limb was not improved. No side effect or adverse event was observed in association with the treatment methods described. During a telephone follow-up, 6 months after the end of treatment, the patient reported no recurrences of neuropathic pain.

### 2.2. Case 2

Patient 2 was a 64-year-old woman who visited our acupuncture and moxibustion department due to pain and weakness in her right lower limbs. 4 months before her first visit to our acupuncture department, she had suffered from irregulate postmenopausal bleeding, at which she was diagnosed with endometrial carcinoma and had accepted the surgery, combined with chemotherapy and radiotherapy in the hospital. After the comprehensive treatment above, she had got severe neuropathic pain in her right lower limb. Her symptoms occurred with no residual tumor tissue, local tumor recurrence, or metastasis illustrated by pelvic MRI. She began to receive pregabalin (75 mg bid), and pain had got partly controlled. However, she started to complain of dizziness and gait disturbance after taking the medicine. Therefore, she decided to stop taking the medicine due to intolerance to the adverse events of pregabalin. After withdrawal, the pain recurred but the intensity was less than before. She started to visit our department and accepted acupuncture without medication. On her first visit at outpatient, pain intensity evaluation (NRS) was 4 points out of 10.

Subsequently, we performed enhanced acupuncture therapy three times per week, which was composed of two forms of acupuncture. After 10 times of therapy, the pain in the right limb was relieved completely. At 6 months after acupuncture therapy, the pain occurred occasionally but the degree was slight. Since the patient only had minor complaints with respect to occasional pain, we decided to observe her symptoms without any management.

## 3. Discussion

Radiotherapy-related neuropathic pain is a common treatment-related adverse effect in the field of radiotherapy with a high prevalence (31%) among cancer survivors (16). It is often manifested as gradual, persisting, or recurrent episodes and shows distinct clinical characteristics in terms of hypersensitivity symptoms (burning, tingling, and electric shock-like sensation) and hyposensitivity symptoms (numbness and muscle weakness) (17). The symptomatology of RRNP is reliant on the anatomic areas of radiotherapy targeted. The three most commonly involved neural tissues include brachial plexopathy resulting from irradiation for lung or breast cancer, lumbosacral plexopathy after pelvic radiotherapy, and axial neuropathy of the spinal cord following cervical radiotherapy (6).

The pathophysiological mechanisms of RRNP are not yet fully understood. Radiation-induced direct axonal damage and demyelination, nerve compression by indirect extensive radiation-induced fibrosis, and nerve ischemia from microvascular damage are the three key factors involved (18). Pharmaceutical approaches including gabapentin, pregabalin, duloxetine, and tricyclic antidepressive agents are strongly recommended as single agents for first-line treatment in the European Society for Medical Oncology guidance (7). The combination of opioids and adjuvants needs to be carefully dosed because it remains uncertain regarding the risk–benefit trade-off (19, 20). However, the results of most approaches on average could only provide pain relief to less than half of the patients treated (21). In addition, the direct evidence focused on the management of RRNP is limited. There are only a limited number of studies conducted on RRNP (17, 22, 23) that results in the recommendation above which is extrapolated from studies in non-cancer related neuropathic pain. Hence, managing pain adequately for this population remains challenging.

We presented the two cases with RRNP that obtained complete relief after acupuncture. This is the first report to show that acupuncture might release neuropathic pain in patients with cancer after radiotherapy. In patient 1, with a combination of acupuncture and anticonvulsant drugs strategy, the burning and electric shock-like pain in the limb was alleviated in 8 weeks. Patient 2 achieved complete remission of pain through acupuncture in the absence of medication because of the inability to tolerate the adverse events of drugs. Both of them showed no recurrence of pain after stopping interventions and during the follow-up visit. In this study, we applied an enhanced acupuncture strategy that chose the local acupoints at limbs, combining acupoints located at the abdomen and the lumbar. First, the mechanism of the lesion in two cases involves the obstruction of Qi and the blood in the liver, kidney, and spleen meridian. Performing acupuncture at the local acupoints on the above three meridians could regulate the Qi and blood in the meridian and release the pain. Second, according to the theory of meridian, the govern vessel and ren vessel could control all the Yang and Yin meridian. Hence, we applied acupoints at the govern vessel and ren vessel to strengthen the dredging of Qi and blood. Some studies support the effectiveness of this strategy in improving peripheral neural function (26).

An overview of systematic reviews showed that acupuncture is beneficial to cancer survivors with fatigue, insomnia, improved quality of life, nausea and vomiting, bone marrow suppression, menopausal, and CINP (11). In general, many similarities exist between CINP and RRNP in aspects of syndrome and mechanism. Some pilot studies highlighted that the use of local acupoints on toes and fingers is the key factor to the effectiveness of decreasing the intensity of CINP (24). However, we considered that the treatment for RRNP might emphasize the use of proximal segmental acupoints more than local acupoints compared to that for CINP. Although the mechanism by which acupuncture manages RRNP has still not been illustrated, we suggest a possible mechanism based on the mechanism of acupuncture for CINP. First, acupuncture might enhance the perfusion of the vasa nervorum and dependent capillary beds supplying local neurons, which improve the removal of inflammatory factors and reduction of tissue toxicity (13, 25). Second, acupuncture appears to provide a microenvironment conducive to neuroregeneration by stimulating the release of neurotrophin (26).

However, this study is limited because it is a case study. Accordingly, a further study that involves an adequate sample size and appropriate control group is warranted to verify the effect of acupuncture in treating RRNP. Moreover, exploratory research on the mechanisms of acupuncture alleviating RRNP is necessary. In addition, to achieve the optimal outcomes of acupuncture, it is needed to investigate the optimum treatment procedure including stimulation mode, acupoints, and duration.

## 4. Conclusion

We reported two patients with RRNP who showed a good reaction to acupuncture. The results of this study showed that acupuncture might be promising for controlling neuropathic pain after radiotherapy in patients with cancer, especially who were intolerant or unresponsive to medications.

## Data availability statement

The original contributions presented in the study are included in the article/Supplementary material, further inquiries can be directed to the corresponding authors.

## Ethics statement

Ethical review and approval was not required for the study on human participants in accordance with the local legislation and institutional requirements. Written informed consent was obtained from the participant/patient(s) for the publication of this case report.

## Author contributions

YH-z provided the case. ZD-f and YL-s performed the acupuncture. ZD-f and RJ-c wrote the draft. ZS-z and ZK collected the clinical data. YL-s and TC-z reviewed and edited the draft. All authors contributed to the article and approved the submitted version.

## Supplementary material

Supplementary material 1 The procedure of acupuncture in this study.
