# Supplementary material for: Enhanced acupuncture therapy for radiotherapy-related neuropathic pain in patients with gynecologic cancer: a report of two cases and brief review
Source: Front Neurol. 2023 Jun 7;14:1163990. doi: 10.3389/fneur.2023.1163990 (PMC10282125; doi:10.3389/fneur.2023.1163990)
Supplement: Supplementary material 1 — The procedure of acupuncture in this study. [file Data_Sheet_1.docx]

| case | Location | acupoints | Angle when inserting needle | Depth | Procedure |
| --- | --- | --- | --- | --- | --- |
| Patient | limbs | SP06三阴交 | 80-90 | 40 mm | Manual acupuncture: A tailored sterile, stainless-steel needle (length: 50mm; diameter: 0.5mm; QH; Chongqing) was inserted into the described acupoints. After the patients felt the *Deqi* sensation, the needles were removed and re-applied at a 30-degree angle. All needles were withdrawn with clean cotton balls pressed to the skin to prevent bleeding. |
|  |  | SP11箕门 | 80-90 | 40 mm |  |
|  |  | SP12冲门 | 80-90 | 40 mm |  |
|  |  | LR04中封 | 80-90 | 30 mm |  |
|  |  | LR08曲泉 | 80-90 | 40 mm |  |
|  |  | LR10足五里 | 80-90 | 25 mm |  |
|  |  | KI03太溪 | 80-90 | 15 mm |  |
|  |  | BL40委中 | 80-90 | 40 mm |  |
|  | Lumbar and abdomen | GV02腰俞 | 80-90 | 30 mm | Electroacupuncture: The acupuncture needle (length: 40mm; diameter: 0.3mm) was inserted into the acupoints and paired alligator clips from an EA apparatus were attached to the needle holders (GV2-GV3 BL32-BL35 CV4-CV6). Stimulation lasted for 30 minutes with a continuous wave of 20 Hz frequency, pulse width 0.5ms, and intensity of 0.1–2 mA depending on the individual participant’s comfort level. |
|  |  | GV03腰阳关 | 80-90 | 30 mm |  |
|  |  | BL32会阳 | 80-90 | 40 mm |  |
|  |  | BL35次髎 | 80-90 | 40 mm |  |
|  |  | CV4 气海 | 80-90 | 40 mm |  |
|  |  | CV6 关元 | 80-90 | 40 mm |  |

**Supplementary Content**

Table 1: the plan of enhanced acupuncture.

Procedure: both of patient 1 and patient 2 were accepted two kinds of therapy including electroacupuncture and manual acupuncture each time. Electroacupuncture was performed first before manual acupuncture.


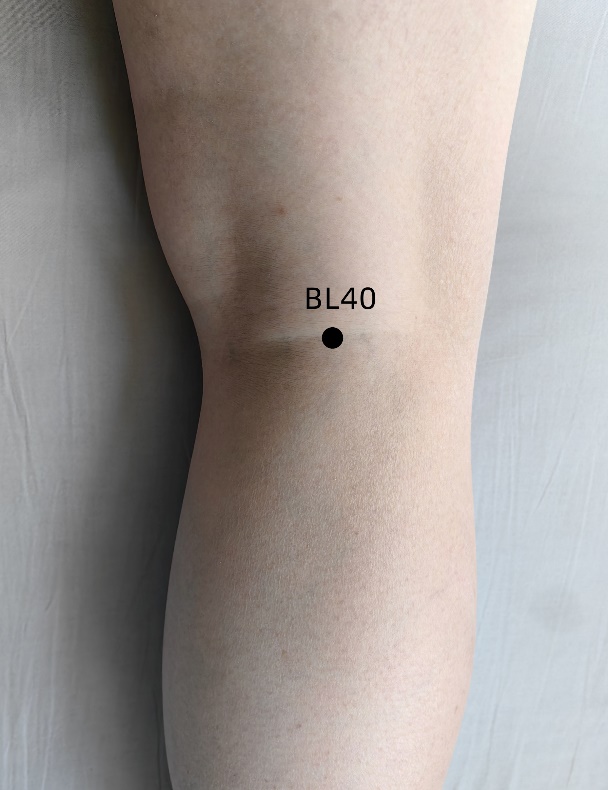

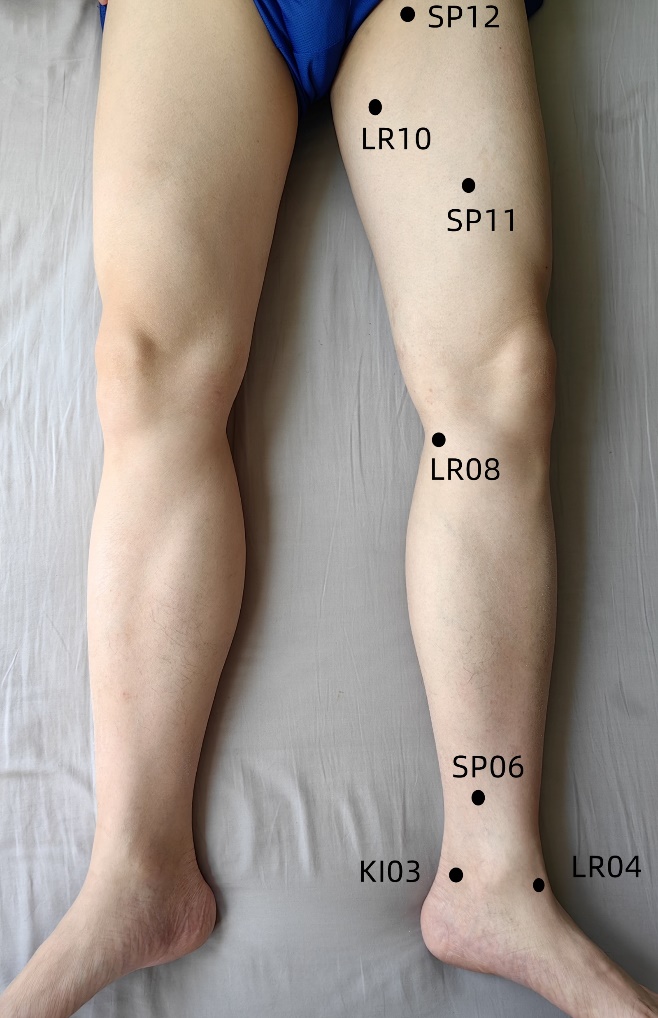


b.

a.


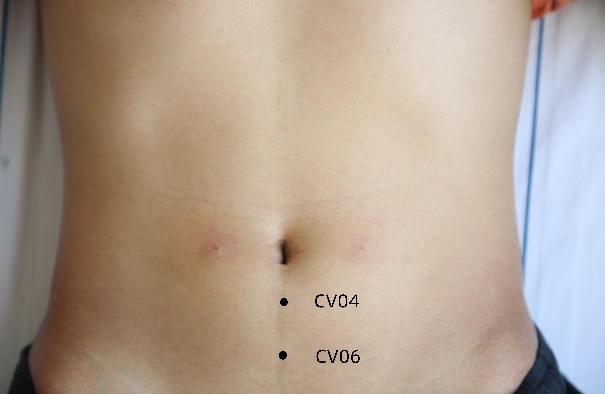


c.


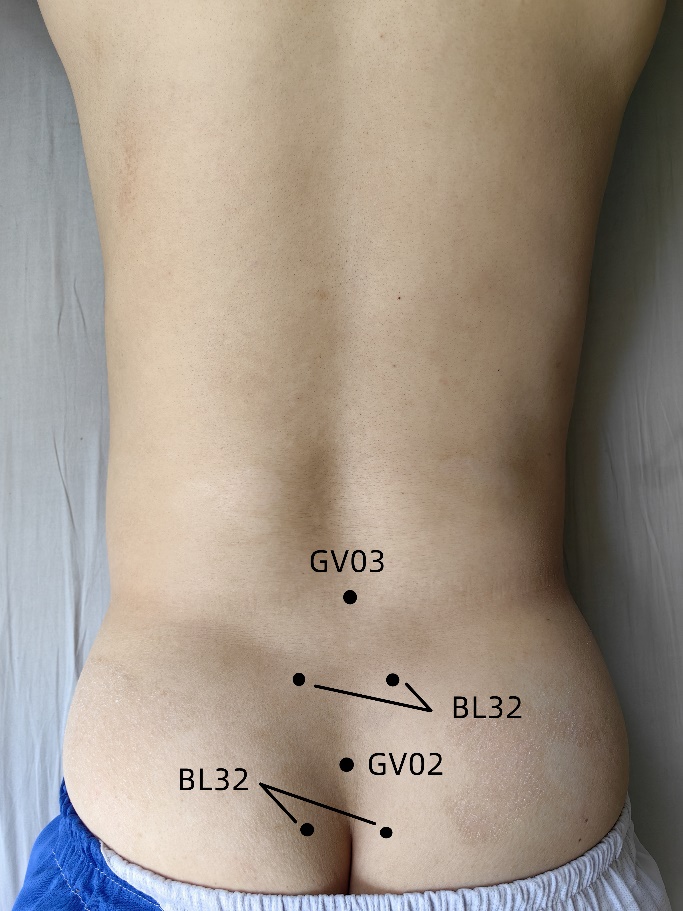


d.

Figure 1: the location of acupoints

a. acupoints in medial side of lower limb b. acupoints in posterior side of lower limb

c. acupoints in abdomen d. acupoints in lumbar
